# Unexpected Adnexal Torsion and Hemoperitoneum Caused by Spontaneous Ovarian Parasitic Leiomyoma: A Case Report

**DOI:** 10.1155/crog/5588436

**Published:** 2025-11-11

**Authors:** Sofia Albuquerque Brás, Mariline D'Oliveira, Raquel Condeço, Filomena Sousa, Bruno Carrilho, Paula Ambrósio

**Affiliations:** Department of Obstetrics and Gynecology, Saint Joseph's Local Health Unit-Maternidade Dr. Alfredo da Costa, Lisbon, Portugal

**Keywords:** abdomen, acute, adnexal torsion, case report, parasitic leiomyoma

## Abstract

Parasitic myomas (PMs) are rare uterine leiomyomas that exist independently of the uterus. They can result from tissue fragments left behind during laparoscopic uterine procedures or, less commonly, develop spontaneously from pedunculated subserosal leiomyomas that detach and revascularize on extrauterine structures. We present a case of a 37-year-old woman with a history of pedunculated subserosal leiomyoma who presented in the emergency department with acute pelvic pain. Imaging revealed a large pelvic mass separated from the uterus and significant hemoperitoneum. Emergency laparotomy was performed, identifying a large left adnexal mass causing adnexal torsion, with partial pedicle rupture as the bleeding source. Histopathology confirmed a benign leiomyoma in the ovarian parenchyma. This case underscores the importance of considering PMs in the differential diagnosis of acute pelvic pain and provides more insight about the etiology of spontaneous PMs, which are rare entities.

## 1. Introduction

Uterine leiomyomas are the most common benign pelvic tumors in women, classified into eight types by the International Federation of Gynecology and Obstetrics (FIGO), with Type 8 encompassing parasitic myomas (PMs). A PM represents an ectopic implantation of a uterine leiomyoma that loses its connection with the uterus and receives blood supply from another source to which it has adhered [1]. These can be spontaneous or iatrogenic. Spontaneous PMs are thought to arise from pedunculated subserosal leiomyomas that adhere to extrauterine structures and survive by obtaining blood supply from them, losing any vascular connection with the uterus [2, 3]. On the other hand, iatrogenic PMs occur as a complication of laparoscopic myomectomy or hysterectomy involving morcellation of the surgical specimen for removal. They arise from fragments unintentionally left in the peritoneal cavity after the morcellation procedure and subsequently attach to and draw blood from adjacent organs to grow [2, 4]. In recent years, the growing use of laparoscopic surgery has led to the emergence of this type of PM, although rarer cases following vaginal or laparotomic surgery have also been reported [2]. PMs can occur in any anatomical structure within the peritoneal cavity and often present with nonspecific symptoms, making diagnosis challenging.

We report a case of a spontaneous PM located in the ovary, causing acute abdomen due to adnexal torsion and hemoperitoneum from partial rupture of the adnexal pedicle.

## 2. Case Presentation

**Figure 1:**
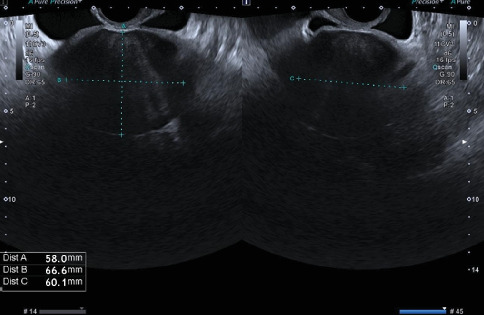
Transvaginal ultrasound, performed in the ultrasound unit of the gynecology department 2 years before, showed a fundal pedunculated subserosal leiomyoma (FIGO 7) measuring 58 × 60 × 67 mm.

**Figure 2:**
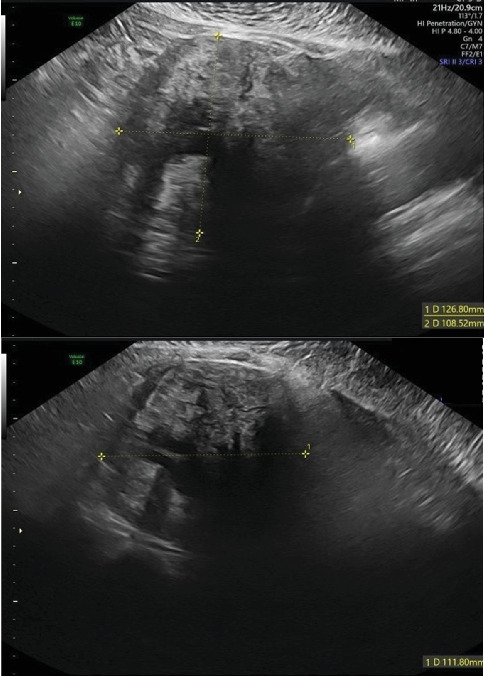
Transvaginal ultrasound, performed in the ultrasound unit of the gynecology department before the surgery, showed a pelvic heterogeneous solid mass, measuring 127 × 109 × 112 mm, with areas of cystic degeneration and without any apparent connection to the uterus.

A 37-year-old multiparous woman (one previous vaginal delivery), with regular menstrual cycles, presented to our emergency department with a 24-h history of sudden-onset pelvic pain. Her last menstrual period had begun 4 days prior, and she was still menstruating. The patient reported no fever, gastrointestinal, urinary, or other associated symptoms. She had a history of an asymptomatic fundal pedunculated subserosal leiomyoma (FIGO Type 7), identified on a routine ultrasound 2 years prior, with a largest diameter of 67 mm (Figure 1). Her surgical history included an appendectomy, and she was not using contraception as she was attempting to conceive. On initial examination, the patient's vital signs were stable; the cervix was laterally displaced to the left without apparent lesions, and there was minimal blood loss; on bimanual examination, a pelvic mass was palpable, extending up to the level of the umbilical scar; there was abdominal rigidity in the left iliac fossa but no guarding. Blood tests showed normal hemoglobin levels (11.2 g/dL), no elevation in inflammatory markers, and a negative Beta hCG. Upon clinical reassessment after the tests, the patient reported a sudden worsening of the pain. Abdominal palpation revealed tenderness, guarding, and peritoneal reaction. Transvaginal ultrasound showed a large hemoperitoneum in all quadrants, a slightly heterogeneous uterus of normal size, and a pelvic heterogeneous solid mass measuring 127 × 109 × 112 mm with areas of cystic degeneration, without any apparent connection to the uterus (Figure 2). Vascularization could not be assessed. The right ovary appeared mobile with no abnormalities, while the left ovary was not identifiable.

**Figure 3:**
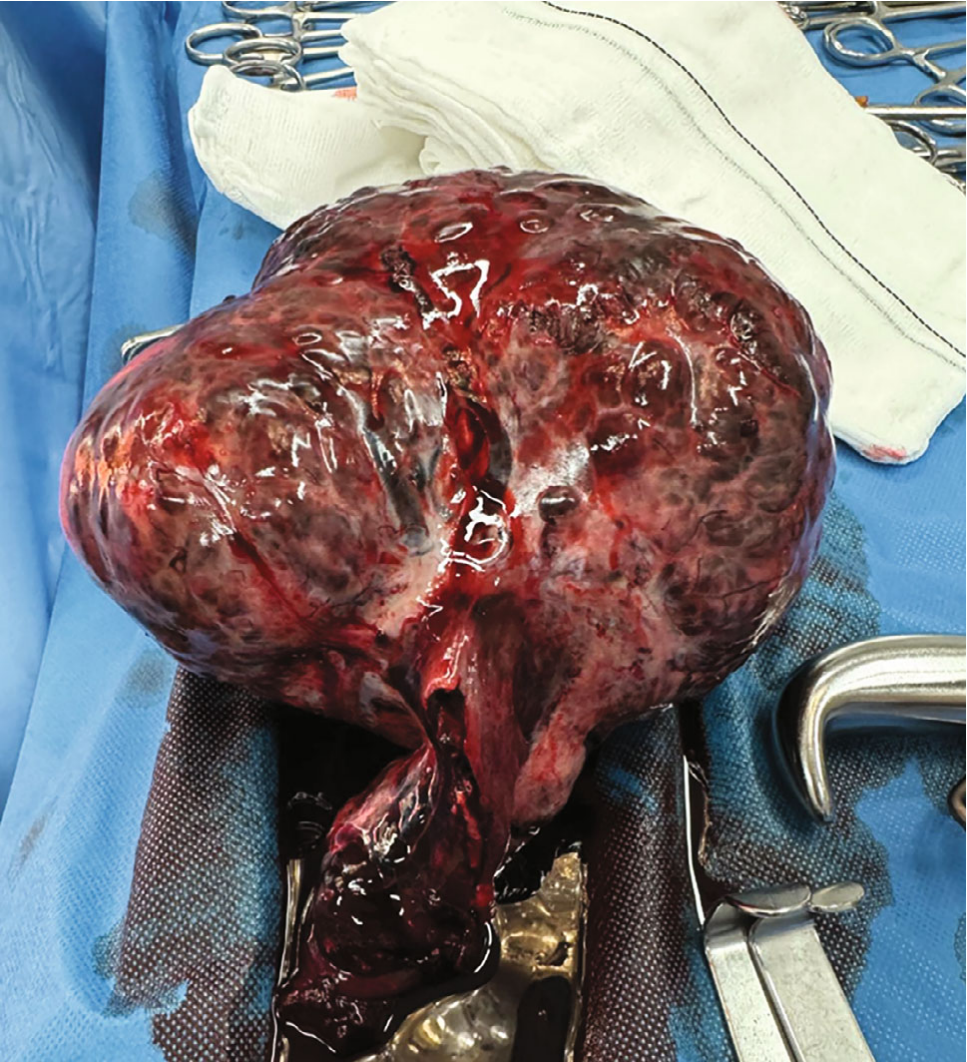
Surgical specimen from adnexectomy.

Given these findings, an emergency laparotomy was performed. Upon entry into the peritoneal cavity, there was moderate hemoperitoneum and a large left adnexal mass causing adnexal torsion was observed. The mass was approximately 15 cm in size and had a bosselated, grayish-brown external surface; a partial rupture of the utero-ovarian ligament was identified as the source of hemorrhage. No abnormalities were noted in the uterus or right ovary. Detorsion and adnexectomy were performed (Figure 3), followed by hemostasis and peritoneal lavage. The estimated blood loss during surgery was 700 mL, and the patient received one unit of red blood cells intraoperatively. Postoperatively, she recovered without complications and was discharged home after 4 days.

At her follow-up appointment, the patient was asymptomatic. Histopathological examination of the 1309 g specimen, which included the uterine tube and ovary (165 × 140 × 100 mm), revealed areas of congestion and hemorrhage consistent with adnexal torsion. In the remaining ovarian parenchyma, a proliferation of spindle cells in a collagenous stroma was documented, with no atypia, necrosis, or appreciable mitotic activity, that expressed Caldesmon and Desmin focally and were negative for SF1 and Inhibin. These findings confirmed the diagnosis of leiomyoma.

## 3. Discussion

While uterine leiomyomas are the most common benign pelvic tumors in women, PMs represent a rare subtype and can pose diagnostic and treatment challenges.

PMs were first described by Kelly and Cullen in 1909 [5]. Since then, numerous cases have been reported in the literature, most within the last few decades, coinciding with the increased use of minimally invasive surgery. Recent studies have proposed an iatrogenic origin for PMs, suggesting that the accidental seeding of fibroid fragments during morcellation in laparoscopic myomectomy could explain their pathogenesis [6]. Additionally, some cases have also been reported following myomectomy via laparotomy [7] or even after a cesarean section [8]. Although less common, there are also reports of PMs developing spontaneously in women with no prior history of uterine surgery [4, 9–15]. In our case, we present a woman with no history of uterine surgery, who had a previously documented FIGO Type 7 leiomyoma on transvaginal ultrasound 2 years earlier. This supports the theory that spontaneous PMs may arise from pedunculated subserosal uterine myomas that detach from the uterus and establish a new blood supply by adhering to extrauterine structures [2, 4, 9].

PMs can adhere to any anatomical structure within the peritoneal cavity, including the peritoneum of the pelvic or abdominal wall, omentum, pouch of Douglas, small intestine, colon, and other less common sites. Patients may be asymptomatic or present with nonspecific signs and symptoms, influenced by the size and location of the myoma [2, 10].

Several atypical presentations have been reported in the literature, including ileal hemorrhage [16], ureteral obstruction [17], pseudo-Meigs' syndrome [18], pulmonary thromboembolism [19], gluteal mass [20], intestinal torsion [21], small bowel obstruction [22], bowel perforation [23], portal hypertension with subsequent heart failure [24], and inguinal mass [25].

PMs may also localize to the ovaries, as observed in our case. Abdel-Gadir et al. reported a case of a spontaneous PM causing elevated inhibin B and secondary amenorrhea [14]. Stebbins et al. described a woman who presented to the emergency department with sudden-onset severe pelvic pain mimicking acute appendicitis, later diagnosed as adnexal torsion caused by a PM, without hemoperitoneum. However, that patient had a history of three cesarean sections, suggesting a possible iatrogenic origin [8]. To our knowledge, this is the first reported case of acute abdomen due to adnexal torsion with hemoperitoneum caused by a PM.

PMs present an intriguing diagnostic challenge, as they can mimic other pathologies [3]. In this case, the patient's history of a FIGO Type 7 leiomyoma, combined with the ultrasound findings, initially raised suspicion of a ruptured pedicle as the source of hemoperitoneum, although the left ovary could not be visualized on imaging. However, intraoperative findings revealed an adnexal mass with torsion and partial rupture of the utero-ovarian ligament as the source of bleeding, with no evidence of fibroids in the uterus. These findings prompted consideration of alternative diagnoses, including primary ovarian neoplasms. The diagnosis of a PM was not made until histopathological examination of the surgical specimen.

This case report describes a unique presentation and adds further evidence supporting the mechanism of spontaneous PM formation. Although PMs are rare, it is important to consider them in the differential diagnosis of acute pelvic pain, particularly in women with a history of uterine fibroids.

## Data Availability

All the data underlying the case report are available as part of the article.
